# Micro-Photographs in Histology

**Published:** 1876-10

**Authors:** 


### Books Reviewed.

*Micro-Photographs in Histology, Normal and Pathological.* By Carl Seiler, M. D., in conjunction with J. Gibbons Hunt, M. D. and Joseph G. Richardson, M. D. Philadelphia: Porter & Coates. London: Macmillan & Co.

We have received the first four numbers of this new publication, each containing four photographs, with brief descriptive letter press. The plates are—I. Section of skin (X 80). II. Epithelioma of Lower Lip (X 80). III. Pavement Epithelium, from a Triton (X 80). IV. Endothelium, from the diaphragm of a Guinea Pig (X 80). V. Elastic Connective Tissue (X 120). VI. Scirrhus of

Mammary Gland (X 180). Non-elastic Connective Tissue from Omentum of a Cat (X 80). VIII. Connective Tissue Corpuscles, from Cornea of a Frog (X 120). IX. Longitudinal Section of Femur—Human Fœtus (X 120). X. Enchondroma (X 120). XI. Hyaline Cartilage (X 180). XII. Transverse Section of Dry Bone (X 80). XIII. Hepatic Cells, from Liver of a Fly (X 80). XIV. Leukæmia of the Liver (X 220). XV. Blood Corpuscules of Man and Ox (X 650). XVI. Fat Cells, from Mesentery of a Cat (X 200).

The real value of these photographic illustrations of histology is much lessened by the excessive claims made for them by Dr. Seiler. He says in his prospectus "This publication is intended to replace the microscope, as far as possible." That "As these pictures are obtained directly from microscopic objects by means of photography" * * "they have the great advantage of being faithful copies of the pictures formed by the lens, and there is nothing produced that is not actually visible in the instrument, thus avoiding the diagramatic character and subjective coloring which are so frequently found in drawings made by means of the camera lucida. In fact, the illustrations used in the lecture-room and found in books are idealized so much as rarely to give an exact impression of the specimen as it really exists."

Now it so happens, that in order to differentiate various tissues in a specimen, it is often necessary to stain it with some color, generally carmine or some shade of red or blue, or both methods may be employed. In these cases the brilliantly colored picture seen in the microscope is far more adequately represented by a well executed colored drawing than by the simple black and white of a photograph, no matter how excellent the latter may be. Nor is this all, as the actinic power of blue light is much greater than that of red, it follows that in the photographic (positve) plate the blue portion of a specimine will appear light while those stained with carmine or aniline red will appear dark in comparison, even though the actual tint of the carmine be lighter than that of the blue. These objections hold good against all photographs of stained or colored preparations, and yet there can be no doubt of the value of *good* photographs of pathological preparations, when they are used with a distinct allowance for the imperfections inherent in this mode of representation. We fear that the student who places implicit faith in Dr. Seiler's glowing prospectus is doomed to far greater disappointment upon comparing these photographs with good preparations, under a good microscope, than he would have suffered had he depended upon the colored drawings of such men as Dr. Beale. And this, not only because of the inherent impossibillity of giving in plain black and white a vivid represensation of a colored object, but because these plates do not, as a general thing, represent the best work of micro-photography. In support of this statement, it is only necessary to call attention to a few of the plates taken almost at random. Plate I, for instance, is labeled "Section of Skin, traversing through the hair-bulbs," but so ill-defined and blotchy is the general appearance that we almost doubt that any one could guess what it was intended to represent. Plate IV. Endothelium, from diaphragm of Guinea Pig, is even worse, and the same is true of Plate V.

Plate XV is a photograph of a slide prepared to show the blood corpuscules of a man and ox side by side upon a micrometer scale, which is photographed with them. This gives a ready means of measuring the positive and comparative diameter of the corpuscules. It is easily seen, even under this comparatively low power for this kind of work (650 diameters,) that the human corpuscules are sensibly larger than those of the ox.

To sum up them, while some of these plates, Nos. III, VII, XII and XV for instances, are very fair, others are very bad, and no great praise can honestly be awarded to any of them. Yet in spite of all its faults, the publication is a valuable one, and we hope it will meet with a liberal support, and that, as Dr. Seiler gains greater skill as a photographer (and uses, perhaps, better lenses,) the quality of the illustraions will steadily improve.

